# Development of a robust hydroponic method for screening of sunflower (*Helianthus annuus* L.) accessions for tolerance to heat and osmotic stress

**DOI:** 10.1038/s41598-021-81072-3

**Published:** 2021-01-18

**Authors:** Muhammad Shehzad, Rao Samran Gul, Saeed Rauf, Wellington Ronildo Clarindo, Jameel Mohammed Al-Khayri, Muhammad Mubashar Hussain, Hassan Munir, Mehdi Ghaffari, Shahid Nazir, Majid Hussain

**Affiliations:** 1 Department of Plant Breeding and Genetics, College of Agriculture, University of Sargodha, Sargodha, Pakistan; 2 Department of General Biology, Federal University of Viçosa, Viçosa, MG 36.570-900 Brazil; 3 Department of Agricultural Biotechnology, College of Agriculture and Food Sciences, King Faisal University, Al-Ahsa, Saudi Arabia; 4 Department of Agronomy, University of Agriculture, Faisalabad, 38040 Pakistan; 5 Seed and Plant Improvement Institute, Agricultural Research Education and Extension Organization (AREEO), Karaj, Iran; 6 Agriculture Biotechnology Research Institute, Ayub Agriculture Research Institute, Jhang Road, Faisalabad, Pakistan

**Keywords:** Physiology, Plant sciences

## Abstract

Hydroponic systems are known to provide a platform for uniform growth conditions until the reproductive stage. However, many plant species, including sunflower, show poor growth and survivability under conventional hydroponic systems due to poor nutrient availability, hypoxia and algal contamination. Thus, we tested various hydroponic systems to select a hydroponic system suitable for screening of sunflower germplasm. Sunflower accessions showed better growth and leaf gas exchange in newly-designed over conventional hydroponic systems. Selected hydroponic systems were further engaged in sunflower accession screening under heat and osmotic stress in a two-pan system (210 cm × 60 cm). Heat stress treatment was applied by growing sunflower germplasm at 42 °C and osmotic stress by adding polyethylene glycol 8000 which decreased the osmotic potential to − 0.6 MPa. There was significant variability among the sunflower accessions for their ability to survive under stress. Accessions such as C-2721 (43%), C-291 (46%) and D-14 (43%) had lower cell membrane injury percentage under osmotic stress and high seedling survivability (60‒80%) under heat stress when compared with susceptible accessions. Moreover, resistant accessions exhibited greater cuticular waxes and root length but lower transpiration losses. The newly designed hydroponic platform proved reliable for the selection of resistant sunflower accessions. Selected parental lines were validated by assessing their hybrids under field trials across two seasons under water and temperature stress during the reproductive phase (autumn). Hybrid H3 obtained by crossing drought and heat resistant parents had the highest seed yield and water use efficiency.

## Introduction

Crop species are often exposed to various abiotic stresses, such as extreme temperature, soil salinity, UV light, hypoxia, metal toxicity and drought. Drought and high temperature are the major factors of global climate change^1^. Approximately 75% of the global land area has been affected by water stress and only 13% of the arable area could be cultivatable free of this abiotic stress^2^. A rise in global temperature due to continued and increasing emission of greenhouse gases may further aggravate the situation^3^.

Abiotic stresses such as high temperature and insufficient water cause reduced yield in various crop species^2,4^. These are interrelated as plants often face multiple stresses simultaneously^2,5^. The interrelationship between these abiotic stresses takes place in several ways. For example, a stress factor can invoke another stress, such as high temperature causing water stress as a result of increasing the evapotranspiration rate^6,7^. Moreover, a stress factor can modulate synergistic impacts; for instance, osmotic stress can increase injury in the presence of heat stress^1^. Empirical plant breeding programs address the issue over simplistically as individual abiotic factors^8^. For this reason, artificially bred varieties are most likely to fail to cope with multiple abiotic stresses under field conditions^9^.

Sunflower (*Helianthus annuus* L.) is an important oilseed crop in regions where it often faces multiple stresses (drought, high temperature), as high temperature also induces water stress due to high evapotranspiration loss^10^. The occurrence of both stresses increases injury and causes greater yield losses^7^. This physiologic change is associated with osmotic stress that results in increased plant injury^8^. Therefore, irrigation is used to ameliorate the hydric stress effects, consequently increasing the production cost where water is scarce. Hence, the development of heat and water stress tolerant varieties has been recommended for sustainable yield in sunflower^1^.

In order to develop new sunflower hybrid varieties, tolerant inbred lines were initially selected for potential use to develop drought tolerant varieties^11–13^. Hydroponic platforms provide an ideal means of screening for stress tolerant inbred lines due to their homogenous growth conditions. It is also an ideal system to study usually hidden parts of the plants such as root length or root biomass which may have direct relevance to various abiotic stress^14,28^. Field experimentation could be subjected to heterogeneous environmental and soil conditions which may influence the accuracy of selection^2^. However, many plant species show poor growth in hydroponic systems due to poor nutrient availability, hypoxia and algal growth^15^. In addition, the success of a hydroponic system depends on maintain a constant pH, electrical conductivity, dissolved oxygen and proper temperature^16^. On the basis of this background, trials were carried out to select a suitable hydroponic system on the basis of better growth, leaf gas exchange characteristics and utilizing this hydroponic system for the selection of heat and osmotic stress resistant sunflower plants from among elite sunflower germplasm lines.

## Results

### Comparison of the hydroponic platforms

**Figure 1:**
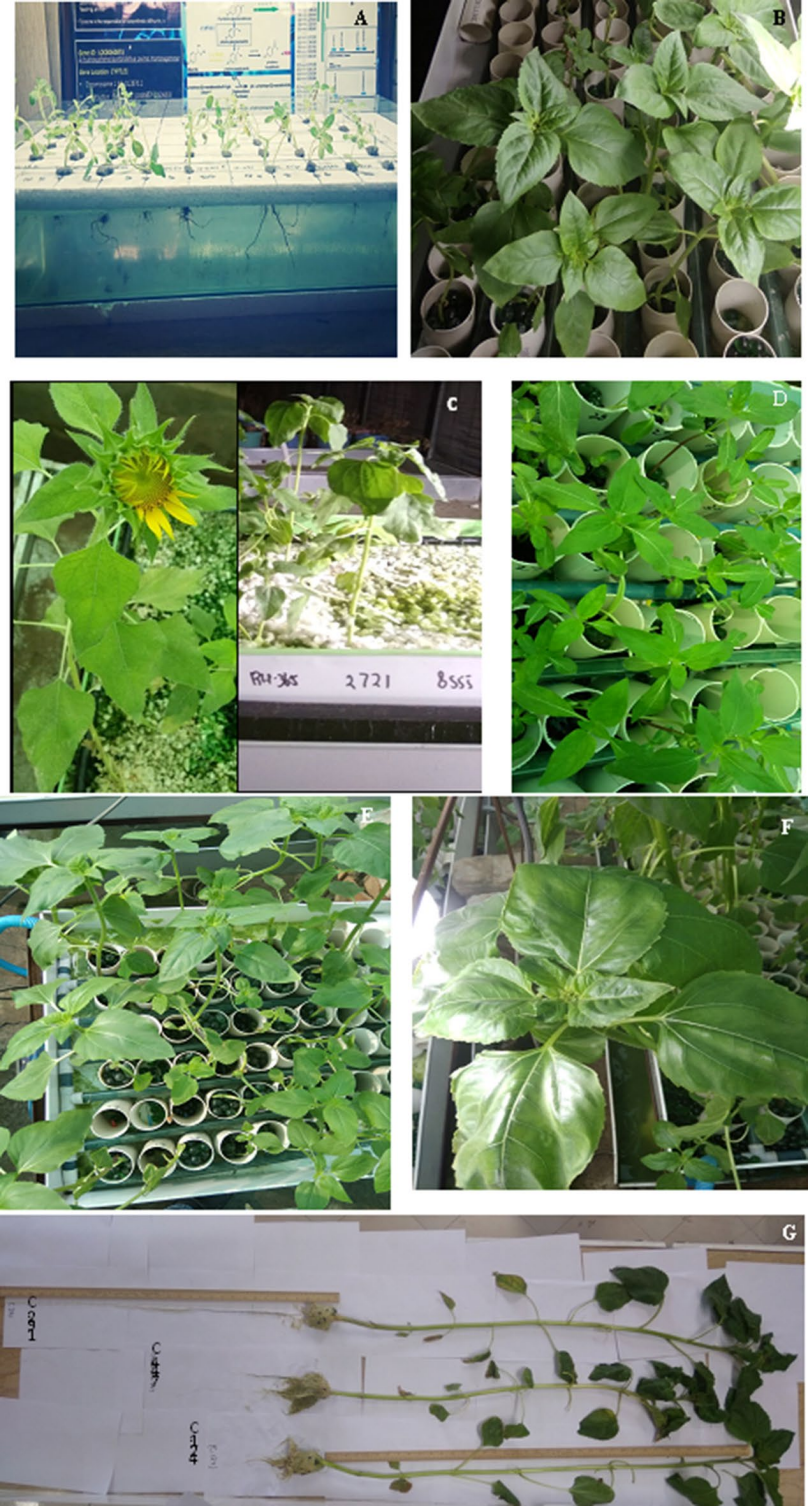
(**A**) Hydroponic platform with roots directly submerged in nutrient media aerated with airstone (HP1) after seedling transplant (15 days after initiation of trial). (**B**) Highly vigrous growth of sunflower (*Helianthus annuus* L.) in two pan hydroponic platform abbreviated as HP3. (**C**) Hydroponic platform HP2 (plant anchored in gravel) contaminated by algal growth (30 days after initiation of trial) and anthesis (65 days after initiation of trial). (**D**) *Helianthus annuus* accessions treated with high temperature in HP3 (Hydroponic platform in which plants anchored in glass ball (8 mm diameter). (**E**) A view of two pan hydroponic platform (HP3) sunflower accessions grown under osmotic stress. (**F**) Plant with disc floret showing the plants approaching their reproductive growth phase. (**G**) Differences among accession for root length i.e. C-291 reaching the root length of more than 1 m in HP3.

Hydroponic systems (HP1, HP2, HP3) were compared in relation to chemical values, overall growth rate of plant material and leaf gas exchange parameters (Tables 1 and 2). HP3, followed by HP2, showed significantly higher survivability of sunflower seedlings as compared with a root submerged platform HP1 (Fig. 1A,B, Table 1). However, the two-pan HP2 platforms with water supplied above gravel showed significantly (P ≤ 0.05) higher hyper hydration and algal growth (Fig. 1C) than HP1 and the two-pan platform HP3. The pH of HP2 fluctuated due to the gravel (Fig. 1C) as the plant substrate; thus, it needed to be adjusted on a daily basis. Using plastic pipes to deliver water over the gravel in HP2 caused algal growth 30 days after the initiation of the experiment (Fig. 1C). Hyper-hydration (glassiness) was also seen in plants close to the water flow (10 cm).

**Table 1:** Mean values of the morphological parameters evaluated for the sunflower germplasm maintained in the various hydroponic platforms.

Hydroponic platform	No. inbred line sown	Survivability%	Hyper hydration%	pH fluctuation	Overall growth rate per week		Leaf area (cm^−1^)		Chlorophyll contents	
					Control	Osmotic stress	Control	Osmotic stress	Control	Osmotic stress
HP1	12	43.21^c^ ± 9.45	6.51^b^ ± 1.93	Stable (5.8 ± 0.1)	0.16^c^ ± 0.10	0.11^b^ ± 0.03	23.56^c^ ± 5.19	17.22^c^ ± 2.66	5.32^b^ ± 1.16	3.51 ± 0.82
HP2	12	80.01^b^ ± 4.52	12.31^a^ ± 2.21	High (5.8‒7.6)	0.26^b^ ± 0.15	0.16^b^ ± 0.05	39.35^b^ ± 7.12	23.52^b^ ± 3.61	10.32^a^ ± 1.31	6.24 ± 1.16
HP3	32	98.34^a^ ± 0.73	0.00	Stable (5.8 ± 0.3)	0.47^a^ ± 0.19	0.28^a^ ± 0.08	94.13^a^ ± 10.35	51.27^a^ ± 4.34	12.16^a^ ± 2.41	7.66 ± 1.14

HP1 = Hydroponic platform with roots directly submerged in nutrient media; HP2 = Two pans with gravelanchoring substrate, HP3 = Two pans with glass beads anchoring substrate.

**Table 2:** Response of the sunflower germplasms for leaf gas exchange parameters under control and osmotic stress treatment in the hydroponic systems.

Hydroponic system	No. inbred line sown	Net photosynthesis rate (µ mol m^−2^ s^−1^)		Transpirational rate (E m mol m^−2^ s^−1^)		Water use efficiency		Stomatal conductance (gs µ mol m^−2^ s^−1^)		Leaf temperature (6°C)	
		Control	Osmotic stress	Control	Osmotic stress	Control	Osmotic stress	Control	Osmotic stress	Control	Osmotic stress
HP1	12	2.13^c^ ± 0.93	1.52^C^ ± 1.05	1.74^c^ ± 0.72	1.18 ^c^ ± 0.36	1.22^a^ ± 0.13	1.29^b^ ± 0.11	0.18^b^ ± 0.05	0.11^c^ ± 0.03	24.32^a^ ± 1.03	25.39^a^ ± 1.14
HP2	12	4.18^b^ ± 0.74	3.14^B^ ± 1.23	3.92^b^ ± 1.01	2.52 ^b^ ± 0.76	1.07^a^ ± 0.11	1.25^b^ ± 0.13	0.29^b^ ± 0.19	0.21^b^ ± 0.08	25.34^a^ ± 0.94	25.82^a^ ± 1.13
HP3	32	7.34^a^ ± 2.19	4.88^A^ ± 1.64	7.23^a^ ± 1.39	3.52 ^a^ ± 0.81	1.02^a^ ± 0.14	1.39^a^ ± 0.18	0.67^a^ ± 0.16	0.42 ^a^ ± 0.0	25.27^a^ ± 1.10	25.62^a^ ± 1.01

HP1 = Hydroponic platform with roots directly submerged in nutrient media; HP2 = Two staged with gravel anchoring substrate, HP3 = Two staged with glass beads anchoring substrate.

The highest growth rate of the sunflower plants was obtained in HP3 (Fig. 1D,E,F) and HP2 (Table 1). However, HP3 had a significantly higher growth rate of sunflower plants than in other platforms (Table 1). HP3 exhibited almost twice the growth rate of HP1 and a 70% higher growth rate over HP2. Leaf gas exchange parameters (Pn, E, g_s_) of HP3 were the highest, except for WUE and leaf temperature (Table 2). There were significant decreasing effects of osmotic stress treatment on morphological and leaf gas exchange parameters (Table 1, 2). HP3 showed greater decrease in the evaluated parameters such as leaf area reduced by 46% in HP3, as compared to 27% and 40% in HP1 and HP2 after osmotic stress treatment (Table 1). The evapotranspiration rate decreased by 51% in HP3, compared to 32 and 36% in HP1 and HP2 (Table 2). These trends showed that HP3 has greater potential to distinguish sunflower germplasm against higher stress levels.

### Evaluation of plants grown under heat stress

**Figure 2:**
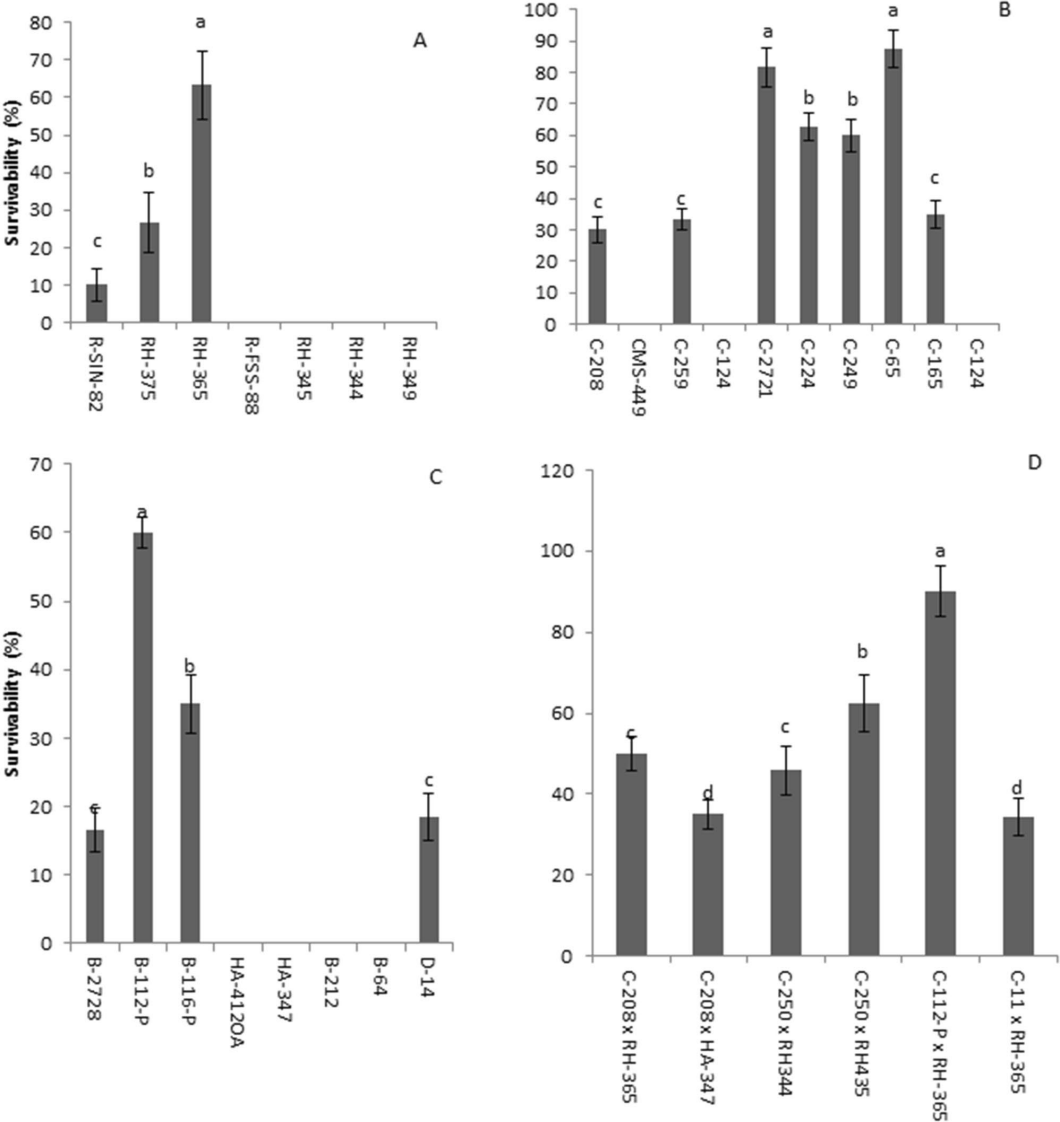
Mean survivability ± standard bar of various sunflower accessions. (**A**) Restorer populations. (**B**) Maintainer. (**C**) Cytoplasmic male sterile lines. (**D**) Cross combinations.

There was a highly significant difference in survivability among the accessions grown under stress conditions (Fig. 1D). Mean survivability of sunflower accessions is shown in Fig. 2. Of the 7 fertility restorer accessions, only 3 survived while the other 4 restorers died after heat stress treatment (Fig. 2A). Fertility restorer breeding accession RH-365 (63%) showed the highest significant mean value of survivability under stress, while cytoplasmic male sterile (CM) accessions (C-2721, C-65, C-249, C-224) had the highest survivability under stress condition (Fig. 2B). C-65 and C-2721 showed significantly higher survivability after heat stress treatment than other CMS accessions (Fig. 2B). Among maintainer accessions, B-112-P, B-116-P and D-14 survived after stress treatment. Accessions B-112-P had the highest (P ≤ 0.05) survivability under stress. Surviving accessions may be regarded as resistant or tolerant, depending upon the mechanism of stress tolerance. Hybrids of accessions C-112-P × RH-365 are both resistant accessions, and had the significantly highest survivability, surpassing the parents in relation to this parameter-hybrid vigor (Fig. 2D). Survivability percentage under heat stress condition was positively correlated with all parameters under study (Table 3). However, it was significantly highly correlated with shoot length (SL) and root weight (RW), while it was significantly correlated with shoot weight (SW) (Table 3).

**Table 3:** Pearson correlation among the morphological parameters of the sunflowers under heat stress.

Parameters	SURV%	LA	RL	SL	R/S	RW
Leaf area (LA)	0.26^NS^					
Root length (RL)	0.30^NS^	0.73**				
Shoot length (SL)	0.54**	0.28^NS^	0.52*			
Root to shoot ratio (R/S)	0.18^NS^	− 0.27^NS^	0.00^NS^	0.12^NS^		
Root weight (RW)	0.59**	0.36^NS^	0.46*	0.52*	0.47*	
Shoot weight (SW)	0.43*	0.67**	0.68*	0.74**	− 0.21^NS^	0.48*

*Significant to P ≤ 0.05. **Significant to P ≤ 0.01.

**Figure 3:**
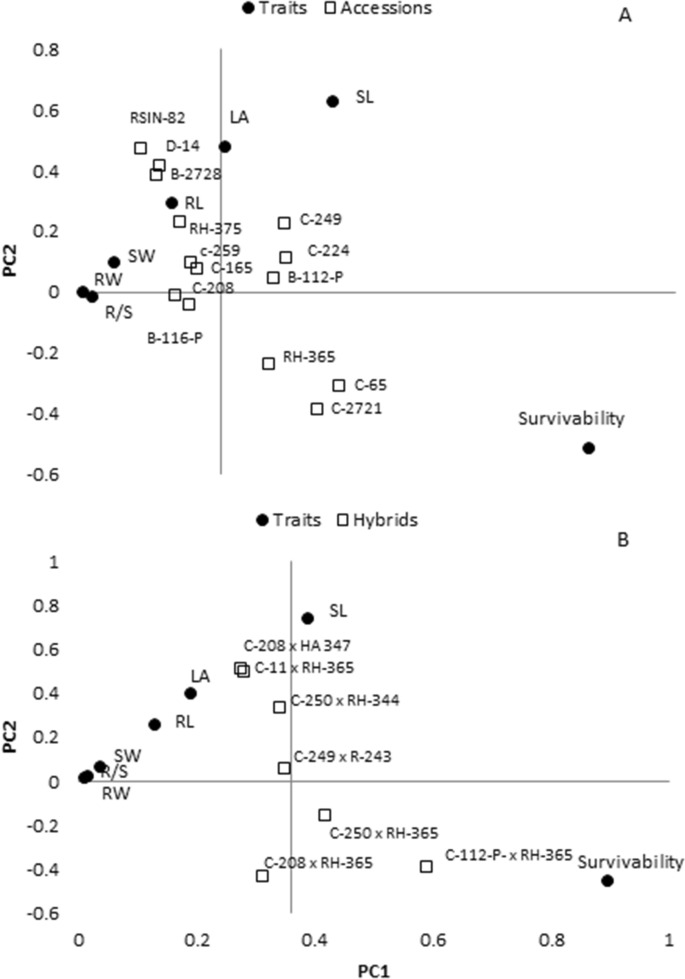
.Genotype + Genotype × parameters,i.e. shoot weight (SW g), shoot length (SL cm), root weight (RW g), root length (RL cm) leaf area (LA cm^2^), root to shoot ratio (R/S) biplot analyses (**A**) sunflower accessions by parameters, (**B**) hybrids by parameters, PC1 = principle component 1 and PC2 = Principle component 2.

Biplot analysis showed that B-116-P was close to the middle of both axes and thus this accession had good performance across several parameters (Fig. 3A). C-2721, C-65 and RH-365 accessions clustered and had the highest survivability (Fig. 3A). C-65 had the highest survivability (88%), RW (3.52 g) and root length (RL, 36.5 cm). D-14 (71.5 cm^2^) had the highest leaf area (LA). C-259 had better SL (82.1 cm) and SW (7.7 g). B-116-P had the highest root shoot ratio (R/S) (0.64) and RW (2.57 g) (Fig. 3A).

### Biplot analysis

Of hybrids is shown in Fig. 3B. Accession combinations C-112-P × RH-365 had the highest survivability (90%) under heat stress, while C-249 × R-243 had the highest value for the parameters i.e. LA (80.1 cm^2^), RL (24.25 cm), RW (1.4 g) under heat stress condition (Fig. 3B). C-250 × RH-344 had the highest SW (8.32) and better RL (24.12 cm) (Fig. 3B).

### Evaluation of the germplasm accessions of sunflowers in water stress

Cuticular waxes (CW) had significantly (P ≤ 0.05) negative correlation with cell membrane injury percentage (CMI) (Table 4). There was a significantly positive correlation between CW under nonstress conditions and CW under water stress. CW under water stress also had positive correlation with SW. The CMI also had negative correlation with root length. Excise leaf water loss (ELWL) had significant negative correlation with RL, SL, RW and SW (Table 4).

**Table 4:** Pearson correlation among morphological parameters of the sunflowers under osmotic stress regime: cell membrane injury (CMI%), excise leaf water loss (ELWL) cuticular waxes under control (CWµ g g^−1^),cuticular waxes under stress (µ g g^−1^) (CW2), root length (RL (cm)), shoot length (SL (cm)), root weight (RW g^−1^) and shoot weight (SW g^−1^).

Parameters	CMI	ELWL	CW	RL	SL	RW	SW
ELWL	0.20^NS^						
CW1	− 0.64**	− 0.11^NS^					
CW2	− 0.04^NS^	0.26^NS^	0.33*				
RL	− 0.34*	− 0.46**	0.24^NS^				
SL	− 0.07^NS^	− 0.39*	0.15^NS^	0.76**			
RW	− 0.15^NS^	− 0.50**	0.28^NS^	0.50**	0.70**		
SW	− 0.16^NS^	− 0.48**	0.38*	0.58**	0.82**	0.95**	
R/S	0.14^NS^	− 0.32^NS^	− 0.28^NS^	0.16^NS^	0.16^NS^	0.51**	0.29^NS^

*Significant to P ≤ 0.05. **Significant to P ≤ 0.01.

**Figure 4:**
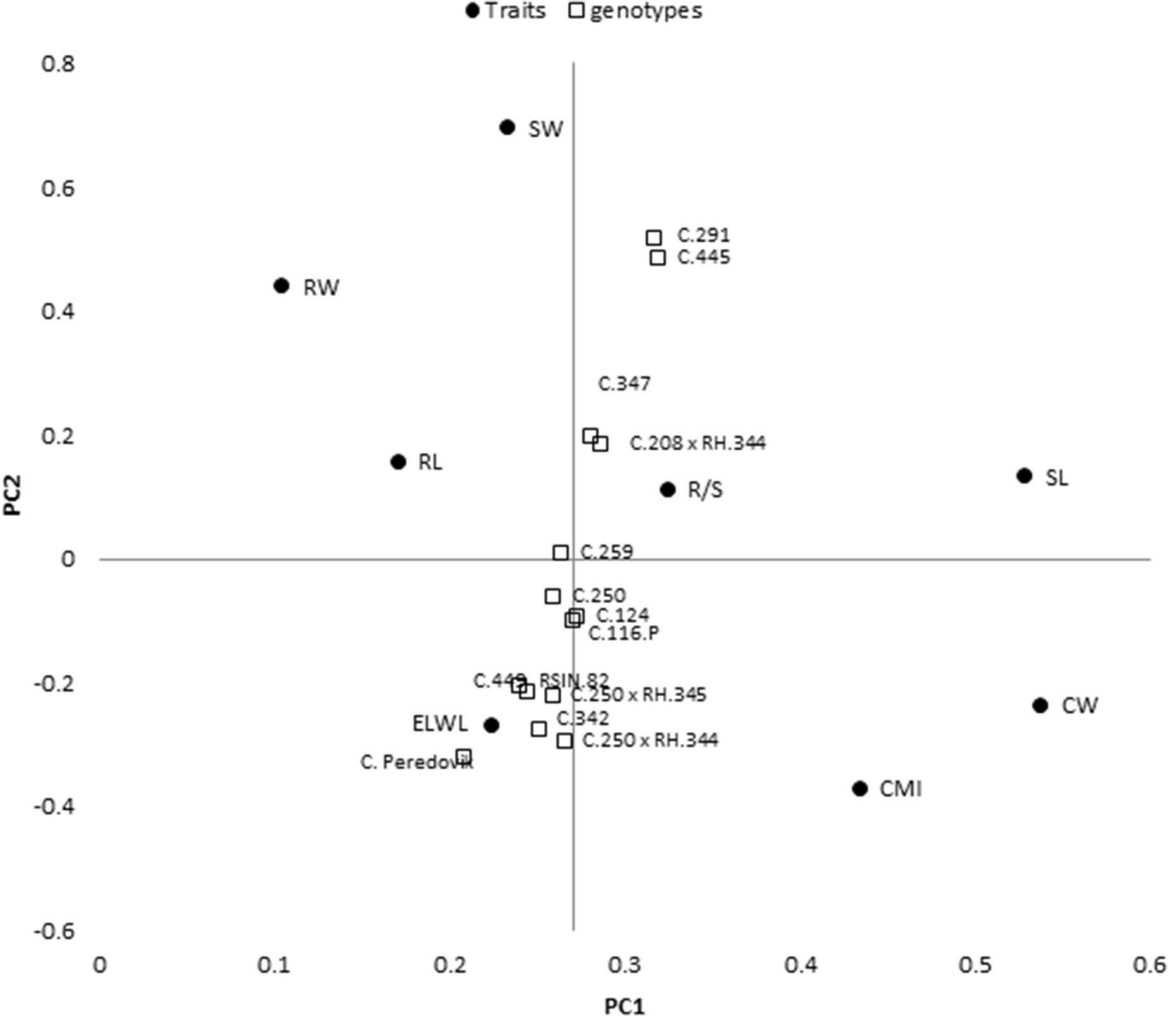
Genotype + Genotype × parameters, i.e. cell membrane injury (CMI), cuticular waxes (CW µg g^−1^), excise leaf water loss (ELWL), shoot weight (SW g), shoot length (SL cm), root weight (RW g), root length (RL cm) leaf area (LA cm^2^), root to shoot ratio (R/S) biplot analyses of sunflower accessions and hybrids under PEG induced osmotic stress (− 0.6 MPa), PC1 = principle component 1 and PC2 = Principle component 2.

Biplot analysis under PEG induced osmotic stress (Figs. 1E, 4) showed that accession C-291 (Fig. 1G), was the most promising genotype under osmotic stress condition due to its highest values for RL (43.12 cm), CW (770 µg g^−1^), RW (31 g) and PH (86 cm). Accession C.445 had the highest RW (39 g) and SW (67 g). Accession combinations C.250 × RH-344 (0.77) and C.208 × RH-344 (0.62) showed the highest R/S. Accession C-259 (20%) followed by C-291 (22%) and C-250 (24%) had the lowest excise leaf water loss (Fig. 4).

**Figure 5:**
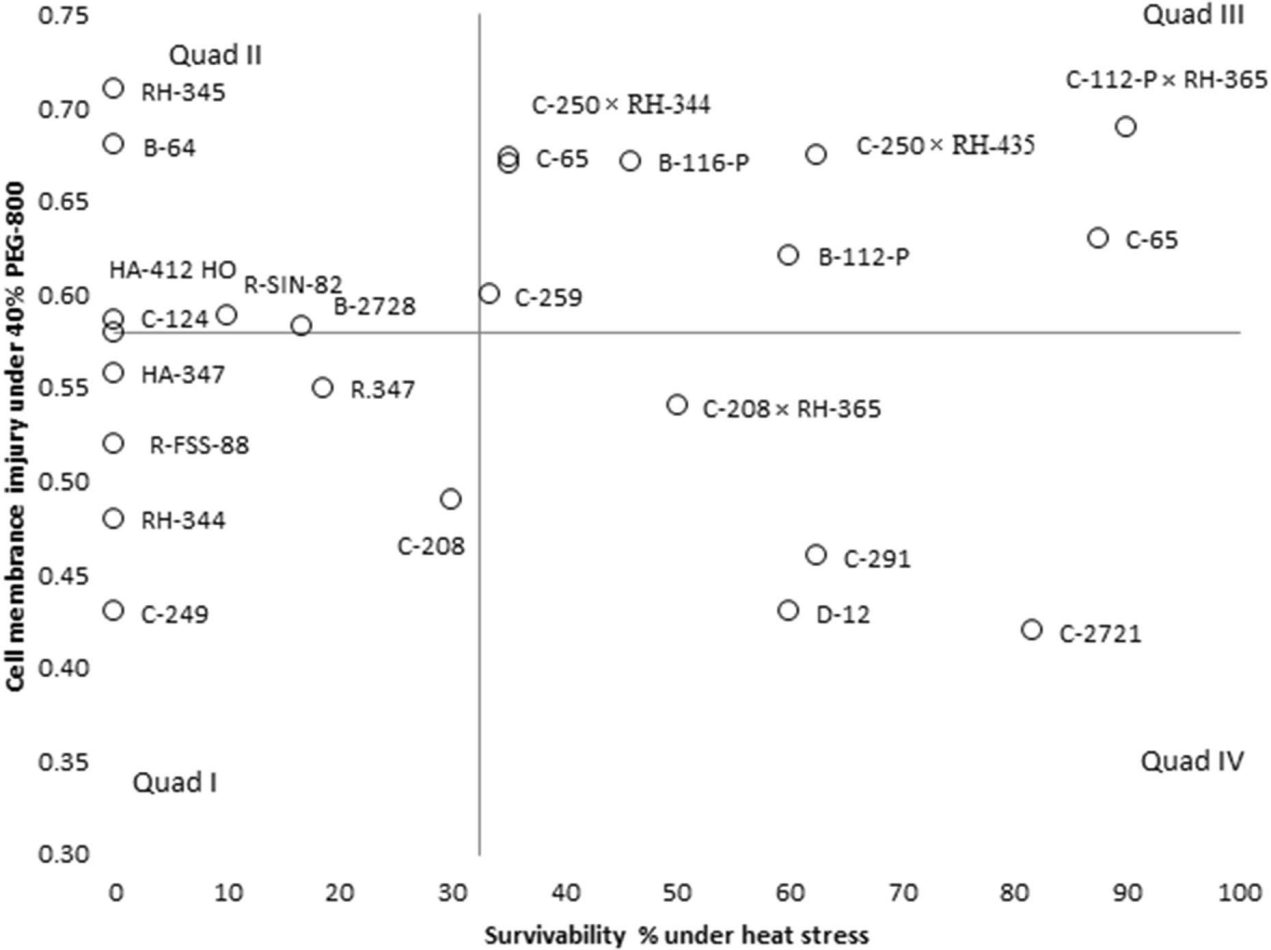
Relationship of survivability % under heat stress with 40% PEG induced cell membrane injury.

Cell membrane injury due to 40% PEG was plotted to survivability under heat stress for the discrimination of accessions against both osmotic and heat stress (Fig. 5). Accessions C-2721, D-12 and C-291 genotypes had simultaneous resistance for osmotic and heat stress (Fig. 5, Quadrate IV). Accessions C-65, C-112-P × RH-365 and B-122 genotypes had good survivability not only under heat stress but had higher CMI (Fig. 5 Quadrate III), and C-208, C-249 and RH-344 were grouped due to lower CMI and survivability under heat stress (Fig. 5 Quadrate I).

**Figure 6:**
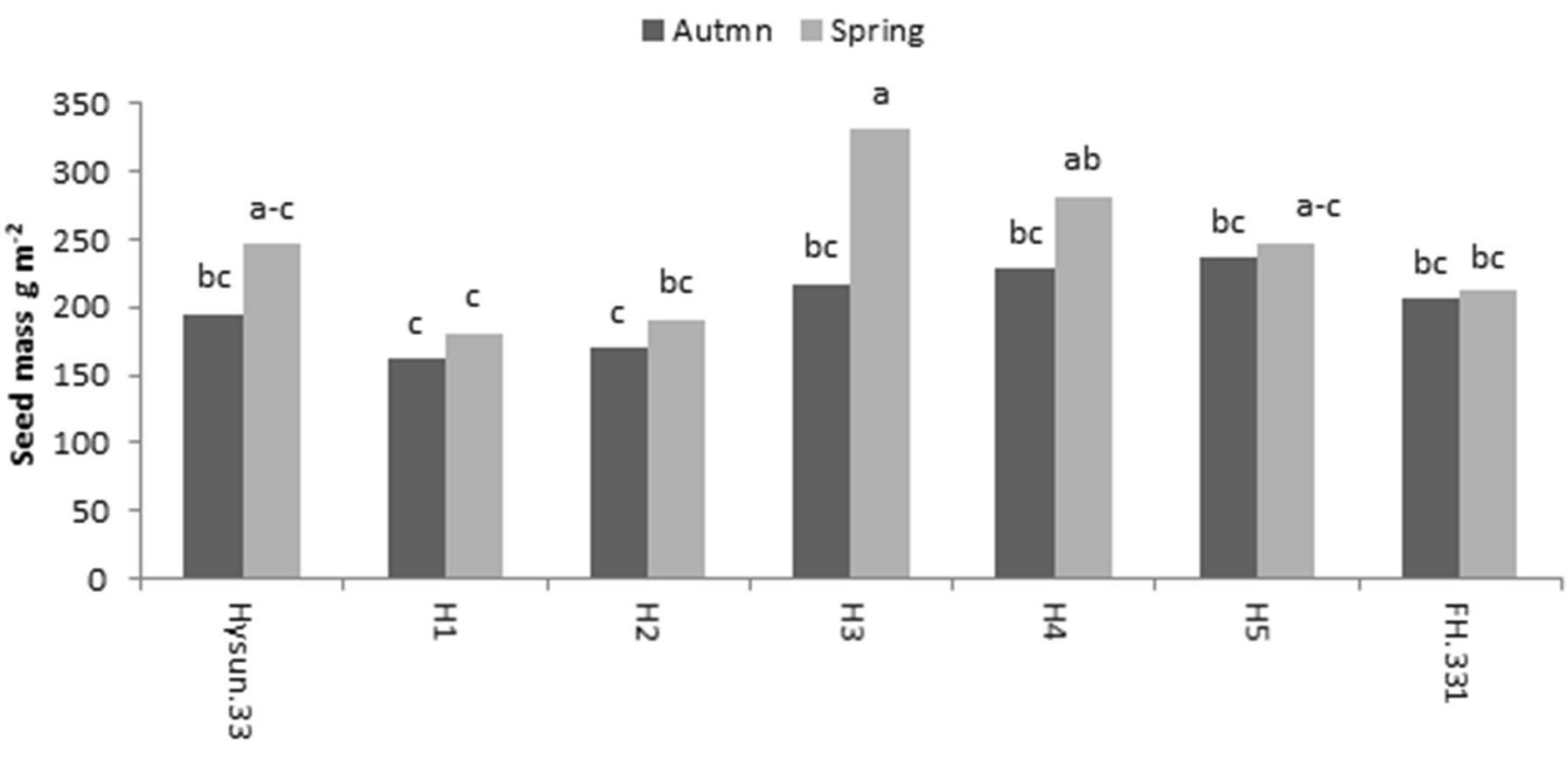
Seed mass (g m^−1^) of sunflower hybrids varied for drought and heat tolerance averaged over locations within spring and autumn seasons.

Seed mass (g m^−1^) of sunflower hybrids is shown in Fig. 6. Hybrid H3 obtained by crossing heat and drought tolerant female and male parents had the highest seed mass, which was 8% higher during the autumn season and 45% higher during spring over commercial checks. Susceptible hybrids (H1, H2) had significantly (P ≤ 0.05) lower yield seed mass than the resistant hybrids (Fig. 6). Resistant hybrids had 30% (autumn) and 40% (spring) yield advantage over susceptible hybrids. Hybrids H3, H4 and H5 are excellent candidates for future commercialization under target conditions.

**Figure 7:**
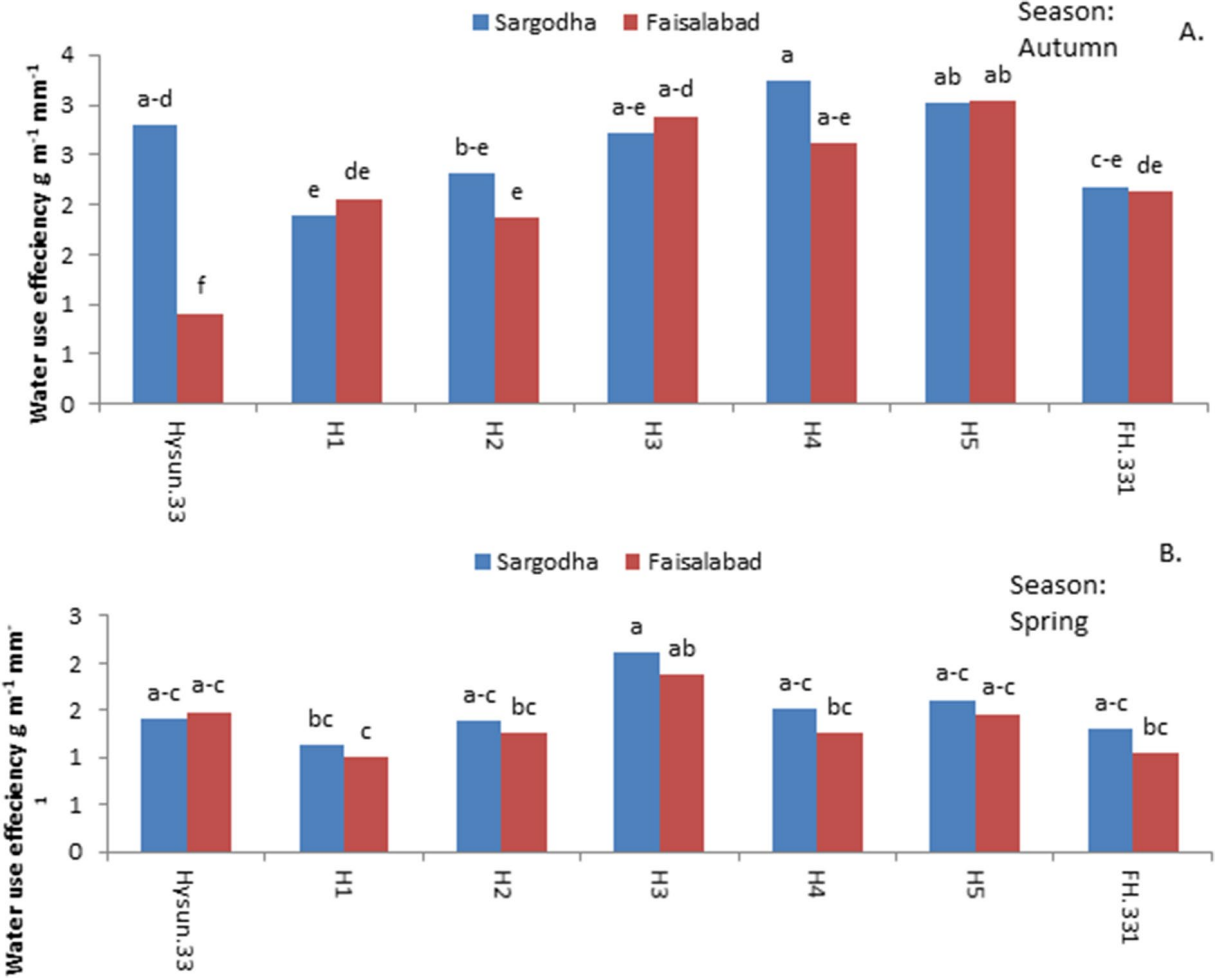
Water use efficiency (g mm^−1^) of sunflower hybrids varied for drought and heat tolerance during (**A**) Autumn and (**B**) Spring seasons.

Hybrid H4 had the highest water use efficiency (WUE) during autumn at the Sargodha site (Fig. 7a). It showed 30% significantly (P ≤ 0.05) higher WUE than commercial checks and 29% significantly (P ≤ 0.05) higher than all susceptible hybrids including commercial checks. Hybrid H3 had significantly (P ≤ 0.05) the highest WUE during spring at both locations (Fig. 7b) which was 50 and 29% higher than commercial checks at the Sargodha and Faisalabad sites, respectively.

## Discussion

Heat and drought are major production constraints and are considered as major abiotic stresses that were widely dispersed across the globe^1,9^. Screening based on sunflower germplasm was an important step to identify resistant accessions, which may subsequently be used in breeding programs. Generally, field experiments failed to provide homogenous conditions and pot experiments may not provide soil and space to obtain full genetic potential of a genotype^2^. According to Conn et al.^15^ and Watson et al.^28^, hydroponic platform overcomes the drawback of the screening methods used here, allowing to check the algal growth, hypoxia and the nutrient availability. These advantages were important to optimize the experimental conditions to sunflowers germplasm, and different hydroponic platforms were compared and optimized for screening purposes.

The newly designed HP3 platform gave the best results to promote improved sunflower growth compared to the conventionally screened HP1 platforms. Based on this, the HP3 platform may be able to improve the growth of other species due to water flow across the roots, enhancing aeration and nutrient availability. The hydroponic results of using HP3 were validated in field conditions where hybrids obtained from crossing of selected heat or drought resistant parents were evaluated under targeted conditions during autumn and spring seasons. Resistant hybrids had enhanced yield and higher WUE than susceptible hybrids, indicating the effectiveness of selection for abiotic stress resistance. Previous studies have utilized the solution culture approach for screening for traits such as salinity and metal toxicity; however, the results have not been validated in pot experiments and under field conditions^30,31^. Other studies have demonstrated that multiple screening methods (solution culture, pot experiments) were more effective in the discrimination of salinity resistant and susceptible germplasm^32^. Poor validation of hydroponic screening trials for abiotic stress in field trials may be attributed to suboptimal growth conditions such as the presence of hypoxia and pH fluctuations. These conditions were addressed in our study resulting in the currently designed hydroponic culture protocol based on choosing appropriate substrate and optimum water flow to the roots. It has been reported that incorporating a substrate such as sand and gravel for root anchoring increases the efficiency of germplasm screening in a hydroponic system^33^. However, the present study has shown that gravel may affect the pH and was replaced by glass balls. Screened traits, such as cuticular waxes and root length, act as a buffer against yield reduction under drought and heat stress conditions and consequently increases WUE^8,13^. Resistant hybrids had higher yields over susceptible hybrids during spring, which coincides with the reproductive phase and heat stress. Therefore, drought and heat stress may have increased the injury due to the sensitive reproductive phase water stress of susceptible germplasm; whereas, the resistant parents screened for tolerance to these types of stresses exhibited better performance under stress conditions^10^.

Plant survivability under stress is an important measure of heat tolerance in sunflower, and has been exploited by plant breeders to differentiate the germplasm^3,20,21^. Seedling survivability under stress is based on various biochemical parameters such as the higher activity of key enzyme scavenging of the ROS species and protecting the protein structure, chlorophyll stability and low respiration losses^22,23^. Screening trials revealed variability among the sunflower germplasm for survivability under heat stress. Survivability under stress was a non-laborious marker for heat stress tolerance and previous results have shown high heritability of this parameter^24^. Genetic studies showed that this seedling survivability was based on monogenic inheritance that could be traced in their progeny. Therefore, the parameter was recommended for exploitation in screening trials^3^.

Variability for PEG induced osmotic stress was also observed among the inbred lines. C-249, C-291, C-208, RH-344 and C-2721 had less cell membrane injury (CMI), which were considered as genotypes tolerant to water stress. Therefore, these genotypes may be used to develop drought tolerant hybrids. Correlation analysis showed a negative relationship among CMI, CW and RL and a positive relationship between CW and RL, indicating that genotypes with lower CMI also have higher CW and RL. CW is a protective layer on the leaf which is known to protect against heat and water stress^8,13^. There was some indication of increased CW and RL under water stress and thus the genotypes showed higher accumulation of CW under osmotic stress. Greater root length was also considered a preferable parameter under water stress and genotypes with longer roots tend to avoid water stress for an extended duration^2^. The genotype with longer roots may be able to extract residual moisture from within root zone more successfully than other accession. In maize and wheat, ‘opportunistic’ genotypes that maintain stomatal conductance and net photosynthesis rate even under dehydration are considered to be more productive than drought-sensitive genotypes^25,26^. Accessions such as C-291 and C-445 may be dependent on resistance mechanisms such as higher CW and RL, which may not be able to conserve water but may be able to extract water from a deep soil profile^2^.

Generally, sunflower accessions respond differentially to both heat stress and PEG induced osmotic stress as there was no evident relationship between the parameters such as cell membrane injury at 40% PEG and survivability under heat stress condition, showing that these two parameters are independent of each other. However, some inbred lines and hybrids tend to show good performance under osmotic and heat stress. Accessions such as C-2721, D-12 and C-291 exhibited simultaneous resistance to osmotic and heat stress (Fig. 5). Various osmolyte accumulations such as sugar and heat shock provide resistance against both stresses^27^.

## Materials and methods

### Plant material

Sunflower seeds of various accessions from the Department of Plant Breeding and Genetics, University of Sargodha (Table 5) were used to screen against osmotic and heat stress. Germplasm accessions were selected based on their agronomic traits such as better combining ability for hybrid breeding, oil content and oil quality (Table 5). Elite germplasm accessions were selected based on their potential use in the development of heat and drought resistant hybrids. Salient features of various accessions are given in Table 5.

**Table 5:** List of accessions used in experiments.

Sr #	Accession	Traits	Salient feature
1	C-208	Inbred line with good agronomic value with high oil contents% and better combining ability for hybrid breeding	Cytoplasmic male sterile (CMS)accessions
2	C-249		
3	C-124		
4	C-259		
5	C-249		
6	C-224		
7	C-65		
8	C-165		
9	C-46		
10	C-449	High oleic acid (80%) fatty acid line responsive to high temperature	
11	C-2721	Mid oleic acid (60%) line inbred line selected from 2721 population	
12	B-116-P	High oleic acid line (80%) selected from Pervenent variety	Germplasm CMS maintainer accessions
13	B-112-P	High oleic acid line (80%) selected from Pervenent variety	
14	HA-412OA	High oleic acid (80%) USDA line	
15	HA-347	High oleic acid (80%)USDA line	
16	B-2728	Mid oleic acid line (60%) selected from 2728 population	
17	B-212	Maintainer line with better agronomic traits	
18	B-64		
19	D-14	Inbred line obtained through interspecific crossing *Helianthus annuus* × *Helianthus*	
20	R-SIN-82	Promising fertility restorer line in hybrid breeding	Male fertility restorer accession
21	RH-365		
22	R-FSS-88	Drought tolerant restorer lines	
23	RH-345	High oleic acid (80%) restorer line	
24	RH-349		
25	RH-344		
26	RH-375		
27	C-208 × RH-365		Accessions combinations (Hybrids)
28	C-208 × RH-347		
29	C-250 × RH344		
30	C-250 × RH435		
31	C-11 × RH-365		
32	C-112-P x RH-365		

Seeds were planted in plastic trays containing a mixture of sand with 10% peat moss and thinned to a single seedling 10 days after germination. The seedlings were maintained at 25 ± 2 °C under 16-h daylight (800 µ mol m^−2^ s^−1^). Six plants of each accession were evaluated in each replication for each hydroponic system (HP1, HP2, HP3). The accessions comprised cytoplasmic male sterile lines, fertility restorer lines and hybrids obtained from crosses between inbred lines (Table 5). The chosen inbred lines and hybrids had good agronomic parameters (oil content, α-tocopherols, fertility) and combining ability.

### Hydroponic platform

Three hydroponic platforms differing in air supply were evaluated. For platform 1 (HP1 Fig. 1A), seeds of 12 sunflower pre-germinated accessions (10 days after germination) were transferred to hydroponic containers (90 × 30 cm). Six seedlings of each accession were anchored in holes on styrofoam sheets with roots submerged in the Hoagland nutrient medium^16^ adjusted to a pH of 5.8 and maintained in the greenhouse at 25 ± 2°C under 16-h photoperiods provided by 60 W LED cool bulbs (600 µmol m^−2^ s^−1^). A 40 L of nutrient solution was added to each replication. A solution of 5% polyethylene glycol (PEG MW 8000) was added to the nutrient medium to achieve an osmotic stress of -0.6 MPa.

For HP2, continuous nutrient medium flow was implemented by installing a submersible pump with a flow rate (2000 L h^−1^). A two-pan system (90 × 30 cm) was developed where the lower pan contained nutrient medium (40 L) and the upper pan contained 40 kg of white gravel forming a 5-cm substrate to anchor the plants. The upper pan contained small holes at 2.5 cm distance from all sides, which act like a sieve to allow nutrient medium to return back to the lower pan. Nutrient medium was supplied continuously, from the lower pan to the gravel pan, through plastic pipes running over the gravel. Seedlings (10 days after germination) of 12 accessions were planted in the gravel pan. The physical environment of HP2 was the same as the HP1, along with control. The medium was changed after 14 days and a pH of 5.8 maintained.

### Hydroponic platform (HP3)

Two trials were separately conducted in the greenhouse to screen germplasm under heat and drought stress. Hydroponics systems were installed with some modifications due to pH fluctuation, algal growth and hyper hydration. Two paned systems (210 cm × 60 cm) were installed (Fig. 1B). Small holes (each 2.5 cm^−1^) were made in the upper pan to allow nutrient medium to return back to the lower pan. Plastic cups (5 cm diameter × 10 cm height) were fixed in the upper tray to keep each plant’s roots separate. There were 6 cups in each row, 32 rows within each hydroponic system and a single accession for each row. Each cup was filled with 8 cm layer of glass balls (8 mm diameter) as the substrate to the hold the plants in place. Each cup was connected to a water supply pipe by small plastic connecter pipes (4 mm I.D.) at the lower base by drilling a hole in the cup. The lower pan contained a submersible water pump with a capacity of 3500 Lh^−1^ which circulated water from the lower to the upper pan continuously. The lower pan contained 200 L of Hoagland solution^16^, pH 5.8 and was rechecked and adjusted as required on a daily basis. The medium was changed after 10 days until the termination of trials i.e. floral bud initiation stage (R1 Fig. 1F).

### Evaluation of physiochemical parameters in response to stress:

For heat stress trials, the temperature was increased gradually from 25 °C to 40 °C at 2°C day^−1^ increments. Greenhouses received natural sunlight supplemented with artificial light supplied by 60 W LED cool bulbs (800 µ mol m-^2^ s-^1^). Water stress was induced when the seedlings reached 18 days of age. PEG (MW 8000) was added to induce a water stress regime to achieve osmotic stress of -0.6 MPa, as compared to the control where no PEG was added. Stress treatments continued until the plants reached reproductive stage, producing floral buds 42 days after sowing.

Different parameters were evaluated in response to exposing the plants to stress factors. Plant survivability was determined on a daily basis by calculating the number of plants that survived after initiation of heat stress (42 °C) until the count became stable for 7 days. Root length (cm), shoot length (cm), shoot weight (g), root weight (g) and leaf area (cm^2^) were determined at the floral bud initiation stage under all stresses and hydroponic systems to assess plant vigor. The shoot weight and root weight were determined using an analytical balance, whereas the length-based parameters i.e. root length and shoot length were determined by a measuring tape and leaf area was by leaf area meter (Model CI-202, Camas, USA).

Fifteen-day old leaves (days counted as a leaf borne in apical tissues), tagged at similar nodes from top to ensure the same age, were used to evaluate the cuticular waxes, cell membrane injury, chlorophyll contents and leaf gas exchange. The cuticular waxes were determined according to^13^ where the leaf discs (16 mm diameter) of 5 plants were obtained and the adaxial side was dipped in chloroform (5 ml) for 3 s to isolate the cuticular waxes. Cuticular wax assay was repeated 3 times plant^−1^ by using a new disc each time and the values were averaged over 3 repeats per plant and given in µg g^−1^.

Cell membrane injury (%) was evaluated following the procedures described by^17^ (www.plantstress.com). The protocol was modified to contain 40% polyethylene glycol (PEG) for sunflower species. Leaves were tagged, and 15 day-old-leaves were harvested from each plant, with 5 plants replicate^−1^ at the floral budding (R1) stage. All collected leaves were washed with deionized water and 3 × leaf discs (14 mm) were dipped in 8 ml (40% PEG solution) and placed in screwed glass vials (18 × 150 mm) to induce osmotic stress; for control treatments, leaf discs of the same size were dipped in dH_2_O. Tubes were incubated at 10°C for 24 h in the dark. Leaf discs from both treatments were washed with dH_2_O, and again re-incubated at 10°C for 24 h after adding 8 ml dH_2_O. Electrolyte leakage was determined through a conductivity meter (4510 Jenway, UK) at 25 °C after incubating glass vials in a water bath (WnB10, Memmert, Germany) for 2 h. All test tubes containing leaf disc were autoclaved (ES-215, Tomy, Japan) at 121 °C for 15 min and glass vials were cooled, and total electrolyte leakage was re-determined at 25°C through a conductivity meter. Cell membrane injury was calculated by the formula given by the Blum and Ebercon (17). The procedure was repeated 3 times by using new discs for each plant at 40% PEG and the mean values recorded.

Chlorophyll content was determined on 15-day-old tagged leaves in 5 plants replicate^−1^ at floral budding (R1) initiation stage with the chlorophyll tester meter (Model CL-01, Chlorophyll meter, Hansatech Instruments Ltd., UK). Various leaf gas exchange parameters were determined with the photosynthesis system Model CI-340, Camas, USA using leaves of similar age (15 days counted as leaf emergence from the apical meristem) during the morning at 10:30–12:00. The leaf gas exchange parameters tested were photosynthesis rate (Pn, µmol m^−2^ s^−1^), stomatal conductance (gs, mmol m^−2^ s^−1^), transpiration rate (E, mol m^−2^ s^−1^) and leaf temperature (LT, °C). Finally, water use efficiency (WUE) was calculated as the ratio of PnE^−1^^18^.

### Validation field trials

#### Selection of parental materials and dvelopment of hybrids

Hybrid seed were produced by manually pollinating the CMS lines (carrying male sterility factor in its cytoplasm and thus unable to produce pollen) from R lines (carrying male fertility restore nuclear gene). CMS and R lines were covered with net bags before anthesis to avoid insect pollinators. Pollination was done early in the morning at 7:30 am by transfering pollen by brush over female floral capitula. The process of pollination was repeated every morning until all stigma within the floral capitula withered out (about 5–6 days). Floral capitula were harvested, dried and achenes were threshed, cleaned through a seive and stored in paper bags at 20 °C for use to grow the hybrid crop. Moreover, CMS lines were also maintained by their maintainer inbred lines, while R lines were maintained by self pollination.

Field trials at two locations, Sargodha and Faisalabad, Pakistan, were chosen to validate the response of the selected hybrids during reproductive phase stresses under two seasons. The autumn season had water stress with low temperatures during the reproductive phase. Conditions in the spring season was marked by water stress and terminal phase heat stress during the reproductive phase (Table 6). Both locations have subtropical conditions in a mixed cropping zone of the central Punjab, Pakistan. Total degree days accumulation was 1889 and 1923 during entire hybrids growth cycle at Sargodha and Faisalabad sites, respectively, during the spring season, whereas the hybrids at both locations accumulated 1724- and 1720-degree days during the autumn season.

**Table 6:** Codes of evaluated sunflower hybrids for field validation of screening.

Cross combinations	Code	Female	Male
Hysun.33	Commercial cultivar	–	–
C.2728 × RSIN.82	H1	S	S
C.259 × RH447	H2	HR	US
C.259 × RH 344	H3	HR	DR
C.249 × RH.347	H4	DR	DR
C.250 × RH.344	H5	US	DR
FH.331	Commercial cultivar	–	–

HR = heat resistant, DR = drought resistant, S = susceptible, US = unselected.

Soils at the Sargodha site were characterized as sandy loam soil with pH 7.27 ± 0.12 , EC = 1 ± 0.08, organic matter 6.12 ± 0.23 µg g^−1^, water holding capacity 18% by weight determined through the gravimetric method, content of N 263 ± 9.43 µg g^−1^, P 6.90 ± 0.65 µg g^−1^ and K 184 ± 9.23 µg g^−1^. Soils of the planting sites at Faisalabad are characterized as sandy clay loam, with pH 7.9 ± 0.2, EC = 1.8 ± 0.1, organic matter 6.72 ± 0.23 µg g^−1^, water holding capacity 19% by weight determined through gravimetric method, content of N 264 ± 23 µg g^−1^, P 9.37 ± 0.38 µg g^−1^ and K 232 ± 27 µg g^−1^. The planting date at Sargodha site was 20 August, 2019 for the autumn crop and 18 February 2020 for the spring season crop. Hybrids were planted on 25 August 2019 and 22 February 2020 for the autumn and spring seasons, respectively at Faisalabad site. Planting was done on ridges 75 cm apart and 22 cm plant-to-plant distance. The experimental site was divided into 3 blocks. Each hybrid was sown in 4 lines within each block. Each line was 6 m long. Hybrid seeds were sown with dibbler at depth of 5 cm. Two seeds were sown in each hole, which was later thinned to a single seedling upon germination. Fertility of soil was augmented with inorganic fertilizer (diammonium phosphate) at 60 kg ha^−1^ and Urea (40 kg ha^−1^). During soil preparation and pre-emergence, dual gold herbicide (S-metolachlor) was sprayed to control weed growth. All experimental sites were irrigated with canal water to assure uniform germination; later, trials were grown on natural rainfall. The experiment site at Sargodha received a total rainfall of 100 mm and 160 mm during entire sunflower crop cycle of autumn and spring seasons, respectively. Rainfall was 56 mm and 172 mm at Faisalabad site during entire crop cycle of autumn and spring seasons, respectively. Spring season trials received most of the rainfall during March, i.e. 95 mm and 83 mm at Sargodha and Faisalabad, respectively. Reproductive phase of hybrids received 20 mm and 36 mm of rainfall at Sargodha site during autumn and spring seasons, respectively. Rainfall during reproductive growth cycle of sunflower hybrids at Faisalabad was 25.3 mm and 39 mm during autumn and spring season, respectively. Evapotranspiration losses were estimated by loss of soil moisture content in soil profile (41–80 cm) and root zone (40 cm). Total reproductive evapotranspiration losses were estimated from soil moisture contents starting at the day of floral bud initiation stage and at the final reproductive maturity stage (identified from pale green color of floral capitula) of each hybrid.

At the Sargodha experimental site, the gravimetric soil moisture contents were 13.44% and 12.18% during autumn season, while 12.65% and 11.8% by mass at anthesis and grain filling period during the spring seasons, respectively. Gravimetric soil moisture contents were 13.3% and 12.12% in the root zone (40 cm) at anthesis and at the grain filling stage in the autumn crop, while 12.06% and 11.5% in root zones (40 cm) of spring crop at the Faisalabad site. Soil moisture content was multiplied by bulk relative density to determine volumetric moisture contents and further multiplied with soil depth to determine moisture contents in mm. The highest evapo-transpiration losses were about 5 mm m^−3^ day^−1^ at Faisalabad during the spring season.

Both experimental sites were protected against the insect pests although during the autumn season no pesticides were applied. Whereas the spring season crop was protected against army worm (*Spodoptera frugiperda* J.E. Smith) by spraying with Lufenuron (Benzoylurea) pesticide at 50 ml 100 L^−1^.

#### Parameter measurements

Seed yield (g m^−1^) were measured by harvesting competitive (having plants on both side) plants in row of 1 m. Floral heads were manually harvested when they changed color to pale yellow at the abaxial side. Heads were dried under shade at room temperature. Achenes were threshed manually, and seeds were placed in paper bags. Achenes were further dried at 35 °C to remove extra moisture for a constant mass (about 10% seed moisture level). Seed masses obtained from 1 m^−2^ row-samples were weighed using a digital balance. Water use efficiency was calculated based:

$$ \begin{aligned} & {\text{Water use efficiency }}\left( {{\text{WUE}}} \right) \, \\ & \quad = {\text{ seed mass }}\left( {{\text{g m}}^{{ - {1}}} } \right) \, /{\text{ total evapo}} - {\text{transpiration during reproductive phase }}\left( {{\text{mm}}} \right) \\ \end{aligned} $$

### Statistical analysis

The experiments were conducted in a randomized complete block design (RCBD) with factorial arrangements and 2 replications. Accessions, hydroponic and stress treatments were considered as factors. Each parameter was averaged over values obtained from 5 plants with 2 replications, 3 hydroponic platforms and 3 stress treatments. Percentage data were transformed prior to statistical analysis using the inverse function of Microsoft Excel 2007. The data were subjected to the analyses of variance (ANOVA) at p ≤ 0.05. The means were compared using multiple comparison test based on Fisher’s least significant difference (LSD) at p ≤ 0.05. Data were statistically analyzed through macros add-in in Excel 2007^19^. Pearson correlation was determined using add-in data analysis function of Microsoft excel sheet. Significance of correlation was determined by finding critical value for correlation coefficient (P ≤ 0.05 and P ≤ 0.01) at n‒2 using two-tailed test. Principle component analysis was used to plot biplot figures to show the relationship between the experimental parameters and to determine the best tolerant genotypes through R software^29^. Analyses of variance for field trials were done under randomized complete design (RCBD) with three factors, i.e., seasons, locations and cross combinations. Means of the hybrids within locations or seasons were compared with LSD test at P ≤ 0.05.

## Conclusion

Hydroponic platforms were established to provide optimized growth conditions for screening of sunflower accessions under stress conditions. A two pan hydroponic (HP3) was designed to increase the nutrient and air circulation in roots of sunflower accessions. Sunflower accessions in HP3 had higher leaf gas exchange parameters and better than growth rate than other hydroponics compared in the study. HP3 was used to screen germplasm under heat and osmotic stress conditions. Heat resistant accessions were selected on the basis of plant survivability while cell membrane injury was used as criterion for selection of osmotic stress resistant accessions. Accessions such as C-2721, D-12 and C-291 had simultaneous resistance for osmotic and heat stress.
